# Supplementary material for: Equiflow: An open-source software package for evaluating changes in cohort composition
Source: PLOS Digit Health. 2026 Apr 8;5(4):e0001342. doi: 10.1371/journal.pdig.0001342 (PMC13061237; doi:10.1371/journal.pdig.0001342)
Supplement: S1 Appendix — (DOCX) [file pdig.0001342.s001.docx]

**First Case Study: eICU**

| **# Initializing Equiflow**  ef = EquiFlow(  data=data_processed,  initial_cohort_label="Initial eICU Patient Cohort",  categorical=['Gender', 'Race/Ethnicity'],  normal=['Age', 'Maximum APACHE Score']  )    **# Add exclusion for patients admitted for cardiac issue**  ef.add_exclusion(  keep=ef._dfs[-1]['non_cardiac_patient'] != 0,  exclusion_reason="heart disease admission",  new_cohort_label="Patients not admitted for heart disease"  )    **# Add exclusion for non-septic patients**  ef.add_exclusion(  keep=ef._dfs[-1]['septic_patient'] != 0,  exclusion_reason="sepsis diagnosis",  new_cohort_label="Patients with sepsis"  )    **# Add exclusion for missing troponin data**  ef.add_exclusion(  keep=~ef._dfs[-1]['max_troponin'].isna(),  exclusion_reason="missing troponin data",  new_cohort_label="Complete troponin data"  )    **# Generate the full flow diagram**  ef.plot_flows(smds=True, legend=True, smd_decimals=1) |
| --- |

Selected Python code utilizing the EquiFlow class to generate the eICU case study flow diagram.

| **# Initialize, categorize, add exclusions, generate plot**  ef_easy = (  EasyFlow(data_processed, title="Initial Patient Cohort")  .categorize(['Gender', 'Race/Ethnicity'])  .measure_normal(['Age', 'Maximum APACHE Score'])  .exclude(  data_processed['non_cardiac_patient'] != 0,  "heart disease admission",  "Patients not admitted for heart disease")  .exclude(  **lambda** df: df['septic_patient'] != 0,  "sepsis diagnosis",  "Patients with sepsis")  .exclude(  **lambda** df: ~df['max_troponin'].isna(),  "missing troponin data",  "Complete troponin data")  .generate(output="eicu_case_study")  )    **# Access generated tables**  print(ef_easy.flow_table)  print(ef_easy.characteristics)  print(ef_easy.drifts) |
| --- |

Selected Python code to generate the identical eICU diagram using the EasyFlow wrapper class.

**Second Case Study: MIMIC-IV**

| **# Loading pre-filtered DataFrames**  first = pd.read_csv("first.csv")  second = pd.read_csv("second.csv")  third = pd.read_csv("third.csv")    **# Instantiate EquiFlow with pre-filtered DataFrames**  eq = EquiFlow(  dfs=[first, second, third],  initial_cohort_label="MIMIC-IV v3.1 Database",  normal=['Age'],  categorical=['Sex', 'insurance', 'ICU Mortality'],  format_normal='Mean +/- SD',  missingness=True,  )    **# Define cohort and exclusion labels**  my_new_cohort_labels = [  "___ ICU visits\nMIMIC-IV Dataset",  "___ ICU visits\nFirst hospitalization\nFirst ICU stay",  "___ ICU visits\nFinal analytic cohort"  ]  my_exclusion_labels = [  "___ excluded\nNot first ICU stay",  "___ excluded\nCreatinine missing or\n ICU stay < 12 h"  ]    **# Generate the flow diagram**  eq.plot_flows(  new_cohort_labels=my_new_cohort_labels,  exclusion_labels=my_exclusion_labels,  smds=True,  legend=True  )    **# View generated table**  table_flows = eq.view_table_characteristics(missingness=True) |
| --- |

Selected Python code for MIMIC-IV case study using prefiltered DataFrames passed directly to EquiFlow
