# Supplementary material for: Equiflow: An open-source software package for evaluating changes in cohort composition
Source: PLOS Digit Health. 2026 Apr 8;5(4):e0001342. doi: 10.1371/journal.pdig.0001342 (PMC13061237; doi:10.1371/journal.pdig.0001342)
Supplement: Attachment — Submitted filename: Plos Digital Health Equiflow review_submission.docx [file pdig.0001342.s002.docx]

This manuscript addresses an important and timely problem; making cohort compositional changes visible during sequential exclusions and the **open-source Equiflow** tool is potentially valuable for transparency and equity in clinical research. However, the paper lacks essential statistical and methodological detail (especially on missing data and SMD calculation), misapplies causal language in the troponin case study, and omits several reproducibility and software-engineering elements necessary for publication. The authors should supply explicit mathematical formulations, sensitivity analyses, and clearer guidance on when the tool is and is not appropriate.

**Major Concerns**

1. Missing-data assumptions and handling
i. The manuscript must explicitly state the assumed missing-data mechanism (MCAR / MAR / MNAR) and the implications of each. Currently the package appears to treat missingness as another category without justifying this from a statistical standpoint.
ii. Provide a dedicated “Missing data assumptions and limitations” subsection in Methods that: (a) gives mathematical notation for how missingness is represented and enters calculations, (b) explains when treating missingness as a category is defensible and when it is not (especially under MNAR), and (c) describes recommended corrective actions (imputation, reweighting, sensitivity analyses) and when to use them. Add sensitivity analyses demonstrating how conclusions change under plausible MNAR/MAR scenarios. (See Rubin 1976; Little & Rubin 2019.)
2. SMD definition and interpretation.
a) Precisely define the SMD used for continuous and multi-level categorical variables (give formulae). If you use a specific extension for categorical SMDs (e.g., based on proportions or a pooled variance analogue), cite and show the formulations.
b) Clarify that Cohen’s d benchmarks (0.2/0.5/0.8) are heuristic and derived for experimental effect sizes; they are not universally appropriate for selection/drift. Provide domain/context guidance (e.g., clinical vs demographic variables).
3. Causal framing in the troponin case study.
   a. The troponin example mislabels the mechanism; measurement availability is a selection/collider issue, not a simple confounder. Conditioning on troponin measurement can induce collider bias. Include a causal DAG illustrating the data-generation / selection process, explicitly identify colliders, and reframe the pedagogic discussion to emphasize selection bias and its consequences. I take this as a front-end and backend development and hence the backend development seems not to have been discussed more in the paper i.e. which variable goes where and the workflow/flow diagram would be essential to give clarity.
4. Statistical testing and p-values.

- The role of p-values must be clarified: are tests descriptive (sanity checks) or inferential? Given the large number of tests across steps/variables, control for multiple testing (e.g., Benjamini–Hochberg FDR) or explicitly justify why correction is inappropriate. Consider de-emphasizing hypothesis testing in favour of effect sizes and confidence intervals for this application.
- Thank you for providing the github repository link <https://github.com/MoreiraP12/equiflow-v2> and I have noticed that the normality assumptions are tested before the data have been inputted. It would be ideal to have the library/package do that initial step of screening and reclassify (normality vs non-normality)

**Moderate concerns**

1. **Figures and tables**: The tables and figures need to be reformatted. Refer to the journal guidelines.
2. **Software engineering / maintenance**: add a “Software availability and maintenance” subsection describing versioning and change policy, CI/test coverage metrics, documentation hosting, and contributor/issue triage plans. Provide community metrics if available.
3. Standardize reference style and date formats; include TRIPOD and PROBAST in the literature review. Make sure every reference has a doi (Again follow the journal templates for submission)
4. Replace informal terms (e.g., “sanity check”) with formal alternatives (“validity check”).
5. Clarify API details (supported input file formats: Pandas DataFrame, CSV, Parquet, etc.), selection rules for top limit categories, and your code example should be in appendix in a well formatted way.
