# Supplementary material for: Equiflow: An open-source software package for evaluating changes in cohort composition
Source: PLOS Digit Health. 2026 Apr 8;5(4):e0001342. doi: 10.1371/journal.pdig.0001342 (PMC13061237; doi:10.1371/journal.pdig.0001342)
Supplement: Attachment — Submitted filename: Response to Reviewers.docx [file pdig.0001342.s005.docx]

**Response to Reviewers**

We thank the reviewers for their thorough and helpful feedback. We have made substantial revisions to address the concerns raised, which we believe substantially strengthen the manuscript. Below, we provide point-by-point responses to each comment, with descriptions of the changes made to the manuscript. All revisions are indicated by tracked changes in the revised manuscript. Please also let us know if there are any concerns you feel we did not address properly or misunderstood.

MAJOR CONCERNS

**SMD definition and interpretation**

**Reviewer Comment:** *Precisely define the SMD used for continuous and multi-level categorical variables (give formulae). If you use a specific extension for categorical SMDs (e.g., based on proportions or a pooled variance analogue), cite and show the formulations. Clarify that Cohen's d benchmarks (0.2/0.5/0.8) are heuristic and derived for experimental effect sizes; they are not universally appropriate for selection/drift. Provide domain/context guidance (e.g., clinical vs demographic variables).*

**Response:** We have substantially expanded the "Statistical Analysis" subsection to address both components of this concern:

We now provide explicit mathematical formulas for SMD calculation for continuous variables and for binary categorical variables. For multi-level categorical variables, we cite and describe the approach by Yang and Dalton, which extends SMD to multicategorical variables by computing the Mahalanobis distance. We also note that Equiflow applies a Hedges' correction factor to all SMD calculations to reduce small-sample bias.

We have added clarification that Cohen's conventional benchmarks (|0.2|, |0.5|, |0.8|) were originally developed for experimental effect sizes in behavioral sciences and may not be universally appropriate for evaluating selection drift in observational cohorts.

We also provide domain-specific guidance, adding that researchers should interpret SMD values with domain-specific context and that the importance of any given effect size is tied directly to the research question and study methodology.

**Missing-data assumptions and handling**

**Reviewer Comment:** *The manuscript must explicitly state the assumed missing-data mechanism (MCAR / MAR / MNAR) and the implications of each. Currently the package appears to treat missingness as another category without justifying this from a statistical standpoint. Provide a dedicated "Missing data assumptions and limitations" subsection in Methods that: (a) gives mathematical notation for how missingness is represented and enters calculations, (b) explains when treating missingness as a category is defensible and when it is not (especially under MNAR), and (c) describes recommended corrective actions (imputation, reweighting, sensitivity analyses) and when to use them. Add sensitivity analyses demonstrating how conclusions change under plausible MNAR/MAR scenarios. (See Rubin 1976; Little & Rubin 2019.)*

**Response:** We appreciate this observation. We have expanded the "Missing Data Handling" subsection in Methods, renaming it to "Missing Data Handling, Assumptions and Limitations" to explicitly address each component of this concern:

(a) We describe how missingness is detected and standardized internally and explain more clearly that the package preserves missingness explicitly rather than implementing deletion or default imputation, allowing researchers to apply context-appropriate imputation if desired. We feel that this is a reasonable approach to allow for researcher flexibility given that we hope that this package will be used in many studies which will require their own different approach to missing data depending on the data and research question.

(b) We provide detailed discussion of MCAR, MAR, and MNAR mechanisms. We clarify that visualizing missingness as a category or excluding missing cases is most logical under MCAR conditions; and that under MNAR, treating missingness as a separate category can possibly be misleading as the missing group may be systematically biased.

(c) We now provide recommendations on missing data handling for researchers to consider when using Equiflow: (1) conducting sensitivity analyses using multiple imputation to assess robustness; (2) explicitly reporting the assumed missing data mechanism and its justification; and (3) acknowledging limitations in generalizability if missingness is likely informative. We note that Equiflow does not implement built-in imputation, and researchers should apply these techniques upstream using established packages if needed.

Regarding sensitivity analyses in the case study itself, the only variable with substantial missingness is troponin measurement itself (the very variable defining the final exclusion step). Imputing troponin values for patients who were not tested would effectively recreate the cohort observed at the preceding stage, rather than providing additional insight. Thus, we did not add MAR/MNAR sensitivity analyses in the case study – please let us know if we are misunderstanding the request here and apologies if so.

Overall, a new limitation has been added to the Discussion noting that:

"The package's default treatment of missingness as a category is most appropriate under MCAR assumptions and may require supplementation with sensitivity analyses under MAR or MNAR mechanisms."

**Causal framing in the troponin case study**

**Reviewer Comment:** *The troponin example mislabels the mechanism; measurement availability is a selection/collider issue, not a simple confounder. Conditioning on troponin measurement can induce collider bias. Include a causal DAG illustrating the data-generation / selection process, explicitly identify colliders, and reframe the pedagogic discussion to emphasize selection bias and its consequences. Include workflow/flow diagram for the tool itself.*

**Response:** We thank the reviewer for this important correction. We have substantially revised the case study discussion to properly characterize the methodological issue:

We reframed the discussion throughout to emphasize selection bias rather than confounding. The Problem Definition section now explains that troponin measurement availability functions as a selection mechanism that can induce collider bias, which hopefully is clearer to readers.

We explain that the decision to order troponin testing may be influenced by both patient characteristics and factors related to the outcome. We have added a causal DAG as a new Figure 1, which illustrates the assumed causal relationships among key variables in the research question. We also added new language in the Discussion section that emphasizes troponin measurement availability’s role in this study.

**Statistical testing and p-values**

**Reviewer Comment:** *The role of p-values must be clarified: are tests descriptive (sanity checks) or inferential? Given the large number of tests across steps/variables, control for multiple testing (e.g., Benjamini–Hochberg FDR) or explicitly justify why correction is inappropriate. Consider de-emphasizing hypothesis testing in favour of effect sizes and confidence intervals for this application. It would be ideal to have the library/package do that initial step of screening and reclassify (normality vs non-normality).*

**Response:** We have significantly expanded the "Statistical Testing" subsection to address each component:

We now explicitly clarify that these statistical tests serve a descriptive rather than an inferential purpose in this context. We agree with your point that SMDs are a more effective way to measure changes in this context but include p-value testing as we assume this to be a feature that many users may desire.

Given that multiple comparisons across variables and exclusion steps can produce falsely significant results, we added language advising caution in interpreting individual p-values, especially in the context of large sample sizes. We emphasize that researchers concerned about multiplicity can apply false discovery rate corrections or focus primarily on SMD magnitudes. Most importantly, we have added the Benjamini-Hochberg test to the Equiflow package, so researchers can use this test if they feel it is necessary.

For normality screening, we have updated the Equiflow package so that Shapiro-Wilk normality testing is performed for samples less than 2000 (guided by Royston’s extension to N=2000 cited in the paper) with the D'Agostino normality test used for larger samples over 2000 to verify distributional assumptions. If normality is rejected (p < 0.05), the package automatically falls back to non-parametric testing. And we have also added these changes to the package itself.

MODERATE CONCERNS

**Figures and tables**

**Reviewer Comment:** *The tables and figures need to be reformatted. Refer to the journal guidelines.*

**Response:** We have made reformatting changes based on reading the journal’s guidelines including changing font size of tables and where captions were located. Please let me know if there are other specific changes we should be making.

**Software engineering / maintenance**

**Reviewer Comment:** *Add a "Software availability and maintenance" subsection describing versioning and change policy, CI/test coverage metrics, documentation hosting, and contributor/issue triage plans. Provide community metrics if available.*

**Response:** We have added a new "Software Availability and Maintenance" subsection in the Implementation Details section that addresses each component.

**Reference style and literature review**

**Reviewer Comment:** *Standardize reference style and date formats; include TRIPOD and PROBAST in the literature review. Make sure every reference has a DOI (Again follow the journal templates for submission).*

**Response:** We have made the following changes:

(a) TRIPOD (Collins et al., 2015) and PROBAST (Wolff et al., 2019) are now cited in the Introduction, thank you for the suggestion.

(b) We now have a DOI for each reference.

**Informal terminology**

**Reviewer Comment:** *Replace informal terms (e.g., "sanity check") with formal alternatives ("validity check").*

**Response:** We have replaced "sanity check" with "validity check" in the Variable Selection section of the Results.

**API details and code formatting**

**Reviewer Comment:** *Clarify API details (supported input file formats: Pandas DataFrame, CSV, Parquet, etc.), selection rules for top limit categories, and your code example should be in appendix in a well formatted way.*

**Response:** We have addressed each component:

(a) Input format clarification: We have added text in the Overview and Installation section clarifying that the package accepts input data as a Pandas DataFrame.

(b) Category selection rules: We have added text in the Variable Specification and Display section explaining that the limit parameter allows users to choose the N categories they would like to display, and those N categories are then selected based on frequency in the initial cohort, with the most N prevalent categories displayed and remaining categories aggregated into an 'Other' group. This feature has also now been finalized within the package code itself.

(c) Code appendix: The code example has been moved from the main manuscript to the Appendix.
