# Supplementary material for: Equiflow: An open-source software package for evaluating changes in cohort composition
Source: PLOS Digit Health. 2026 Apr 8;5(4):e0001342. doi: 10.1371/journal.pdig.0001342 (PMC13061237; doi:10.1371/journal.pdig.0001342)
Supplement: Attachment — Submitted filename: Equiflow Manuscript second revisions.docx [file pdig.0001342.s004.docx]

**Equiflow Manuscript (PDIG-D-25-00774R1)**

This manuscript presents Equiflow, an open-source Python package for generating enhanced participant flow diagrams that track compositional changes in cohort characteristics during data preprocessing. The tool addresses a genuine gap in research transparency by visualizing how exclusion criteria alter demographic and clinical distributions beyond simple sample size reductions. The revision has substantially addressed previous concerns regarding SMD definitions, missing data assumptions, causal framing, and statistical testing.

**Major Concerns Requiring Attention**

1. Statistical Testing Section Contradictions

The manuscript states that statistical tests serve a "descriptive rather than an inferential purpose" but then describes automated hypothesis testing with p-value thresholds (p < 0.05 for normality rejection). This creates conceptual confusion:

- If tests are descriptive, why implement formal hypothesis testing with alpha thresholds?

- The recommendation to "prioritize effect sizes over p-values" conflicts with the package's automated test selection based on normality p-values.

Clarify the philosophical stance. Either (a) remove formal hypothesis testing entirely and rely solely on SMDs, or (b) acknowledge that tests serve a screening function and provide clearer guidance on interpretation.

2. Case Study Generalizability

The case study uses eICU data where the final exclusion step (requiring troponin measurement) reduces the sample from 17,161 to 1,094 (93.6% exclusion). This extreme reduction may not be representative of typical exclusion scenarios. The manuscript would benefit from:

- A second, less extreme case study demonstrating utility in more common scenarios

- Discussion of how Equiflow performs when exclusions are more modest (e.g., 10-20% reduction)

**Minor Concerns**

1. Software Engineering Details

- The manuscript mentions "continuous integration testing via GitHub Actions" but does not report test coverage metrics. For a methods paper, reporting coverage percentage would strengthen confidence in software reliability.

- The statement "typical response times of 1-2 weeks" for issue triage is vague. Consider specifying whether this applies to bug reports, feature requests, or both.

2. Reproducibility

- The case study code is available on GitHub, but the manuscript does not specify the exact package version used. Given that the package is under active development, version pinning is essential for reproducibility.

- The eICU data access requires credentialing; consider providing a synthetic dataset for users to test the package without PhysioNet access.

3. Terminology Consistency

- The manuscript alternates between "selection bias" and "compositional bias" without clear distinction. Consider defining these terms explicitly in the Methods section.

4. Figure Quality

- Figure 2 (code example) appears to be a screenshot or image of code rather than properly formatted code. For a software methods paper, code should be presented in a monospace font with syntax highlighting if possible, or moved entirely to supplementary materials with proper formatting.

5. Literature Gaps

- The manuscript does not cite the RECORD guidelines (Benchimol et al., 2015) for reporting studies using routinely collected health data, which are highly relevant.

- No mention of the PROBAST-AI extension currently under development, which may supersede some recommendations.

**Minor Typographical/Formatting Issues**

1. Reference 7 (STROBE) has an incorrect DOI pointing to a different publication (10.1016/j.ijsu.2014.07.014 is for International Journal of Surgery, not PLoS Medicine).
