# Supplementary material for: Equiflow: An open-source software package for evaluating changes in cohort composition
Source: PLOS Digit Health. 2026 Apr 8;5(4):e0001342. doi: 10.1371/journal.pdig.0001342 (PMC13061237; doi:10.1371/journal.pdig.0001342)
Supplement: Attachment — Submitted filename: Response_to_Reviewers_auresp_2.docx [file pdig.0001342.s006.docx]

**Response to Reviewers**

We thank the reviewer for their thoughtful and constructive feedback on our revised manuscript. We have carefully addressed each comment and believe the manuscript is further strengthened as a result. Below, we provide point-by-point responses to each comment, with descriptions of the changes made to the manuscript. All revisions are indicated by tracked changes in the revised manuscript. Please also let us know if there are any concerns you feel we did not address properly or misunderstood.

MAJOR CONCERNS

**Reviewer Comment:** *The manuscript states that statistical tests serve a “descriptive rather than an inferential purpose” but then describes automated hypothesis testing with p-value thresholds (p < 0.05 for normality rejection). This creates conceptual confusion: If tests are descriptive, why implement formal hypothesis testing with alpha thresholds? The recommendation to “prioritize effect sizes over p-values” conflicts with the package’s automated test selection based on normality p-values. Clarify the philosophical stance. Either (a) remove formal hypothesis testing entirely and rely solely on SMDs, or (b) acknowledge that tests serve a screening function and provide clearer guidance on interpretation.*

**Response:** We appreciate this observation and agree that the simultaneous description of tests as “descriptive” while implementing formal hypothesis testing with alpha thresholds created an inconsistency. After careful consideration, we have chosen to remove the Statistical Testing section and TablePValues class description from the manuscript (and will be removed from the package entirely in the next version). The revised manuscript now focuses exclusively on standardized mean differences (SMDs) as the primary metric for quantifying compositional changes between cohorts, which we believe provides a cleaner and more conceptually coherent framework for this tool’s purpose. SMDs are the appropriate measure for tracking compositional drift, as they are sample-size invariant and directly interpretable as effect sizes.

**Reviewer Comment:** *The case study uses eICU data where the final exclusion step (requiring troponin measurement) reduces the sample from 17,161 to 1,094 (93.6% exclusion). This extreme reduction may not be representative of typical exclusion scenarios. The manuscript would benefit from: A second, less extreme case study demonstrating utility in more common scenarios; Discussion of how Equiflow performs when exclusions are more modest (e.g., 10-20% reduction).*

**Response:** We agree that the extreme reduction in the troponin exclusion step (93.6%) may not reflect all research scenarios, and that demonstrating Equiflow’s utility across a range of exclusion severities would strengthen the manuscript.

We have added a second case study using MIMIC-IV v3.1 data (now presented as Figure 3 and Table 2 in the revised manuscript). This case study demonstrates Equiflow’s utility with more modest exclusion steps: restricting to each patient’s first ICU stay (31% reduction) and requiring complete creatinine data with adequate length of stay (6% reduction). The first exclusion step revealed an insurance SMD of 0.07, driven by a shift from Medicare toward private insurance coverage, demonstrating that Equiflow can surface subtle compositional changes even in modest exclusions. The second exclusion step produced uniformly small SMDs (all ≤ 0.03), showing that Equiflow provides a transparent record of cohort stability when exclusion effects are minimal.

Further, we have added discussion in the manuscript acknowledging the extreme nature of this exclusion step and explicitly noting that Equiflow is applicable scenarios with more modest exclusions (e.g., 10–20% reductions), where subtle compositional shifts may be easier to overlook but can still meaningfully affect study validity. We note that even small, incremental exclusions can compound to produce substantial demographic drift across multiple steps.

MINOR CONCERNS

**Reviewer Comment:** *The manuscript mentions “continuous integration testing via GitHub Actions” but does not report test coverage metrics. For a methods paper, reporting coverage percentage would strengthen confidence in software reliability. The statement “typical response times of 1-2 weeks” for issue triage is vague. Consider specifying whether this applies to bug reports, feature requests, or both.*

**Response:** Thank you for this suggestion. We have updated the Software Availability and Maintenance section to include our specific test coverage information. The test suite comprises 51 tests (41 unit tests and 10 integration tests) executed across Python versions 3.9 through 3.12 via GitHub Actions. We have also clarified the response time language to specify that the 2 week response time applies to initial triage of feature requests, with bug reports prioritized within 1 week for faster resolution.

**Reviewer Comment:** *The case study code is available on GitHub, but the manuscript does not specify the exact package version used. Given that the package is under active development, version pinning is essential for reproducibility. The eICU data access requires credentialing; consider providing a synthetic dataset for users to test the package without PhysioNet access.*

**Response:** We agree on both points. We have updated the manuscript to specify the exact package version used for the case study (Equiflow v0.1.10). Additionally, the GitHub repository now includes a synthetic data generator that produces eICU-like data with realistic demographic distributions and clinical characteristics, allowing users to test the package without requiring PhysioNet credentialing. We have added a note in the manuscript describing this.

**Reviewer Comment:** *The manuscript alternates between “selection bias” and “compositional bias” without clear distinction. Consider defining these terms explicitly in the Methods section.*

**Response:** Thank you for noting this inconsistency. We have added explicit definitions now. We clarify that “selection bias” refers to the broader epidemiological concept of systematic error introduced when the study sample is not representative of the target population, while “compositional bias” refers specifically to shifts in the demographic or clinical makeup of a cohort resulting from sequential exclusion steps. Equiflow is designed to detect and visualize compositional bias, which is one mechanism through which selection bias can arise.

**Figure Quality**

**Reviewer Comment:** *Figure 2 (code example) appears to be just a typed code or screenshot/image of code rather than properly formatted code. For a software methods paper, code should be presented in a monospace font with syntax highlighting if possible, or moved entirely to supplementary materials with proper formatting.*

**Response:** We agree about the code presentation. The code found in our Appendix has now been reformatted using a monospace font with proper formatting and syntax highlighting. The main text references the Appendix for the complete code listing, which contains code for both case studies.

**Reviewer Comment:** *The manuscript does not cite the RECORD guidelines (Benchimol et al., 2015) for reporting studies using routinely collected health data, which are highly relevant. No mention of the PROBAST-AI extension currently under development, which may supersede some recommendations.*

**Response:** Thank you for identifying these important references. We have added a citation to the RECORD guidelines (Benchimol et al., 2015) in the Introduction, as these are highly relevant given that Equiflow is often applied to studies using routinely collected health data such as EHRs. Regarding the PROBAST+AI extension, the extension has been published in the BMJ (Moons et al., 2025). We have added a sentence in the introduction citing PROBAST+AI and explaining that Equiflow complements PROBAST+AI, which emphasizes fairness and data representativeness across its signaling questions.

MINOR TYPOGRAPHICAL/FORMATTING ISSUES

**Reference 7 (STROBE) Incorrect DOI**

**Reviewer Comment:** *Reference 7 (STROBE) has an incorrect DOI pointing to a different publication (10.1016/j.ijsu.2014.07.014 is for International Journal of Surgery, not PLoS Medicine).*

**Response:** Thank you for catching this error. We have corrected the DOI for Reference 7 (STROBE) from 10.1016/j.ijsu.2014.07.014 to the correct DOI: 10.1371/journal.pmed.0040297. We apologize for this typo.
